# Iron stores at birth in a full-term normal birth weight birth cohort with a low level of inflammation

**DOI:** 10.1042/BSR20202853

**Published:** 2020-12-14

**Authors:** Joy Y. Zhang, Jing Wang, Qinsheng Lu, Meizhen Tan, Ru Wei, Gendie E. Lash

**Affiliations:** 1 Division of Uterine Vascular Biology, Guangzhou Institute of Pediatrics, Guangzhou Women and Children’s Medical Center, Guangzhou Medical University, Guangzhou, China; 2 Department of Children Health Care, Guangzhou Women and Children’s Medical Center, Guangzhou Medical University, Guangzhou, China

**Keywords:** cord blood, ferritin, iron stores, neonates, soluble transferrin receptor

## Abstract

Iron stores at birth are essential to meet iron needs during the first 4–6 months of life. The present study aimed to investigate iron stores in normal birth weight, healthy, term neonates. Umbilical cord blood samples were collected from apparently normal singleton vaginal deliveries (*n*=854). Subjects were screened and excluded if C-reactive protein (CRP) > 5 mg/l or α1-acid glycoprotein (AGP) > 1 g/l, preterm (<37 complete weeks), term < 2500g or term > 4000g. In total, 762 samples were included in the study. Serum ferritin, soluble transferrin receptor (sTfR), hepcidin, and erythropoietin (EPO) were measured in umbilical cord blood samples; total body iron (TBI) (mg/kg) was calculated using sTfR and ferritin concentrations. A total of 19.8% newborns were iron deficient (ferritin 35 μg/l) and an additional 46.6% had insufficient iron stores (ferritin < 76 μg/l). There was a positive association between serum ferritin and sTfR, hepcidin, and EPO. Gestational age was positively associated with ferritin, sTfR, EPO, and hepcidin. In conclusion, we demonstrate a high prevalence of insufficient iron stores in a Chinese birth cohort. The value of cord sTfR and TBI in the assessment of iron status in the newborn is questionable, and reference ranges need to be established.

## Introduction

Sufficient iron stores at birth support optimal development of the brain and nervous system during early infancy [1]. However, little attention has been focused on the assessment of iron status at birth because of the belief that normal birth weight, healthy, term infants are born with sufficient iron stores to facilitate growth and metabolism during the first 6 months of life [2].

Assessing iron status is complex, no single iron indicator is sufficiently specific or sensitive to be used alone [3]. Serum ferritin concentrations are recommended as the primary measure of iron status at the population-level, although levels may be affected by inflammation [4]. For neonatal populations, studies have suggested thresholds for iron insufficiency (ferritin < 76 μg/l) [5] and iron deficiency (<35 μg/l) [6], which have been applied in several previous studies [7,8]. But the validity of these markers and thresholds is starting to be questioned [9].

Serum soluble transferrin receptor (sTfR) reflects cellular iron needs, and has been combined with serum ferritin levels to assess iron status. Total body iron (TBI) is calculated on the basis of the ratio of sTfR to ferritin concentrations and estimates iron stores on the basis of body weight [10]. However, this estimation has not been used in cord blood samples to evaluate iron stores in newborns [11,12].

Additional biomarkers of interest are hepcidin and erythropoietin (EPO), which monitor iron hemostasis from different aspects. Hepcidin (hepatic protein) is the master regulator of iron homeostasis, and circulating hepcidin levels are inversely related to iron demands in the body [13]. EPO is the principal hormonal stimulator of erythropoiesis and reflects iron utilization. Animal studies have shown that EPO prioritizes available iron for erythropoiesis for the brain, and potentially improves myelination and maturation of the brain [14]. Anemic premature infants with higher plasma EPO levels exhibit improved neurodevelopmental outcomes [15,16].

The present study aimed to investigate the concentrations of serum ferritin, sTfR, hepcidin, and EPO in cord blood samples in a cohort of singleton vaginal birth full-term normal birth weight infants, using C-reactive protein (CRP) and α1-acid glycoprotein (AGP) to screen for both acute and chronic inflammation.

## Materials and methods

### Study design and participants

A total of 854 pregnant women were recruited on the delivery suite of Guangzhou Women and Children’s Medical Center (GWCMC) between December 2016 and December 2017. Infants were included after vaginal delivery with Apgar (appearance, pulse, grimace, activity and respiration) score between 8 and 10 at 1 min after birth. Cord blood clamping and sampling was performed 1–2 min after birth. Delayed cord clamping is not standard practice at GWCMC. Written informed consent was taken from all mothers prior to collection of umbilical cord blood samples. Cord blood samples were kept at 4°C until centrifuged (15 min at 3000×***g***) and serum stored at −80°C until required for analysis. The inclusion criteria were: term (>37 completed weeks) apparently normal pregnancy, singleton, and vaginal delivery. Subjects were excluded if they had CRP > 5 mg/l (*n*=0) or AGP > 1 g/l (*n*=3), were pre-term (*n*=73), term < 2500 g (*n*=5) and term > 4000 g (*n*=11). A total of 762 qualified for analysis in the present study. The present study was conducted according to the Declaration of Helsinki guidelines, and all procedures were approved by GWCMC Ethical Review Board.

### Analytical methods

Serum ferritin, sTfR, hepcidin, EPO, CRP, and AGP were examined by ELISA (CUSBIO CSB-E05187h, CSB-E09100h, CSB-E13062h, CSB-E04538h, CSB-E08617h, CSB-EL017237HU, Wuhan, China) in the Uterine Vascular Biology Laboratory, Guangzhou Institute of Pediatrics, GWCMC. The inter- and intra-assay coefficients of variation were all below 5%. TBI was calculated from the serum transferrin receptor/serum ferritin ratio as follows: TBI (mg/kg) = −[log(R/F ratio) − 2.8229]/0.1207 [9].

### Statistical analysis

Statistical analysis was conducted using PASW® Statistics Version 20.0 (SPSS, IBM® U.S.A.). The distribution of all variables was tested by Kolmogorov–Smirnov. Descriptive statistics (mean and SD; median and interquartile range; frequency and percentage) were determined for all variables. Primary correlations were explored using Spearman’s test (non-parametric). A *P*-value <0.05 was considered statistically significant.

Determinants of cord ferritin levels including gestational age, birth weight, maternal BMI at delivery and paternal smoking status were explored in unadjusted models. The residuals of the multiple linear regression model were slightly deviated from normal distribution; therefore a square-root transformation of cord ferritin levels, birth weight, length, and gestational age was used, the residuals were approximately normally distributed when the regression model was repeated. All tests were two-sided at the 5% statistical significance level, *P*<0.05 being considered statistically significant.

## Results

A total of 854 cord samples were collected, none of the samples had CRP > 5 mg/l but 3 subjects had AGP > 1 g/l, 73 of the infants were delivered before 37 completed weeks of gestation, 5 term infants had a birth weight < 2500 g, and 11 had birth weight > 4000 g. A total of 762 term normal birth weight subjects were included in the present study. The principal maternal and infant characteristics are presented in Table 1.

**Table 1:** Principal maternal and infant characteristics of the subjects enrolled in the study (*n*=762)

Maternal age (years)	30.0 (25, 35)
Primary parity (%)	41.3
Maternal weight at delivery (kg)	64 (54, 74)
Maternal height (m)	160 (154, 166)
Maternal BMI at delivery (kg/m^2^)	25 (21.4, 28.6)
Maternal BMI >30 kg/m^2^ at delivery (%)	6.0
Infant sex – male (%)	54.0
Infant birth weight (kg)	3.3 (2.8, 3.8)
Infant birth length (cm)	50 (48, 52)
*Received university education % (*n*=255)	79.6
*Gestational weight gain (kg) (*n*=349)	14.0 (9.0, 19.0)
*Paternal smoking (%) (*n*=341)	40.1

Abbreviation: BMI, body mass index. Median; interquartile range in parentheses (all such values); used in the case of non-normally distributed variables.

* Data from lifestyle questionnaire.

**Figure 1:**
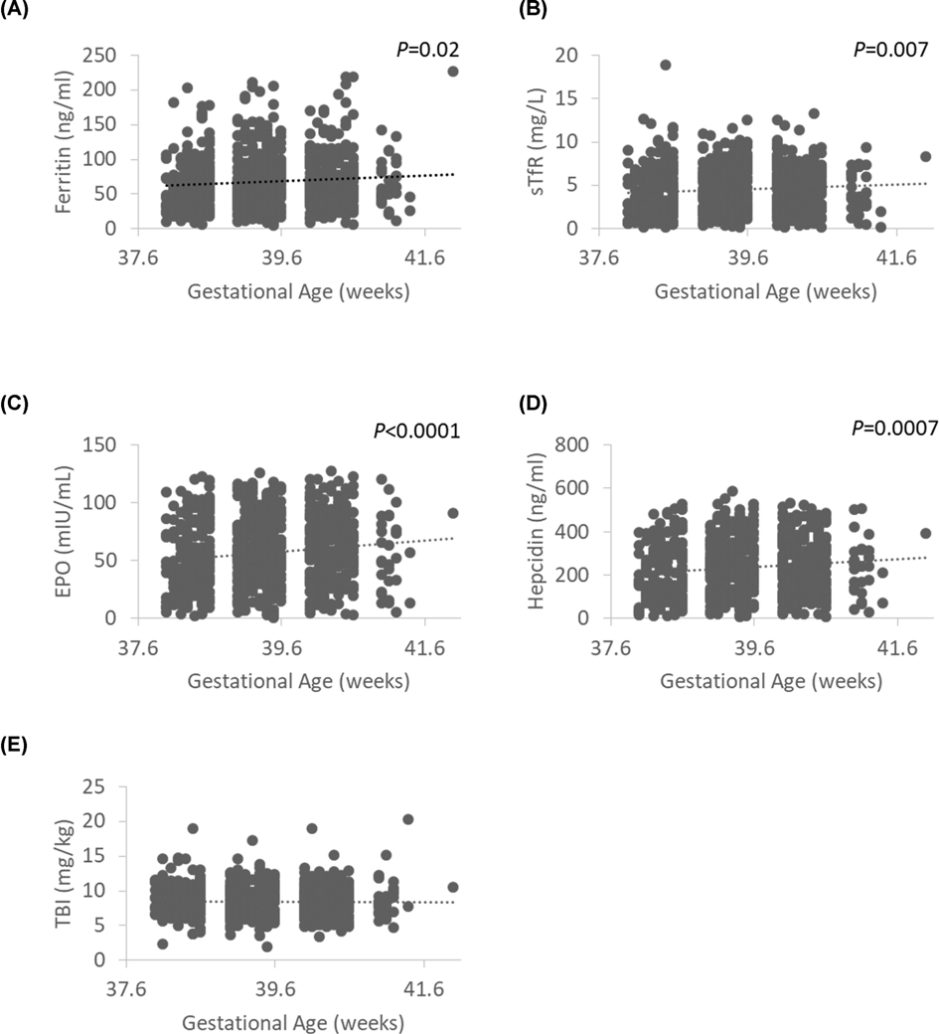
Association between gestational age and different markers of iron stores Association between gestational age (weeks/days) and (**A**) cord serum ferritin (*n*=762), (**B**) cord serum sTfR (*n*=762), (**C**) cord serum EPO (*n*=762), (**D**) cord serum hepcidin (*n*=762), and (**E**) TBI (*n*=762).

The distribution of cord serum ferritin, sTfR, transferrin, hepcidin, and EPO concentrations in the 762 participants are presented in Table 2. We evaluated possible factors in the prediction of cord ferritin levels, including gestational age, birth weight, maternal BMI at delivery, and paternal smoking status (none of the mothers self-reported smoking during pregnancy). Only gestational age correlated with cord serum ferritin levels in term infants in this cohort, with a mean difference per day (95% CI) of 0.5 (0.1, 0.9) μg/l (*P*=0.02; Figure 1A). In addition, gestational age also correlated with sTfR (r = 0.1, *P*=0.007; Figure 1B), EPO (r = 0.1, *P*<0.001; Figure 1C), and hepcidin (r = 0.1, *P*=0.0007; Figure 1D) concentrations. There was no relationship between gestational age and TBI (Figure 1E).

**Table 2:** Distribution of cord serum ferritin, sTfR, TBI estimates, and EPO in the subjects enrolled in the study (*n*=762)

	Mean (SD)	Median (IQR)	Range
Cord serum ferritin (ng/ml)	68.4 (39.7)	61.3 (12.6, 110.0)	4.8–226.7
Cord serum sTfR (mg/l)	4.6 (2.7)	4.3 (0.1, 8.5)	0.1–18.9
TBI (mg/kg)	8.5 (2.2)	8.3 (5.4, 11.2)	1.9–20.2
Cord serum hepcidin (ng/ml)	233.1 (138)	224.4 (16.3, 432.6)	6.9–586.2
Cord serum EPO (mIU/ml)	56.9 (30.2)	55.5 (7.4, 103.6)	0.6–127.0

Insufficient iron stores (ferritin < 76 μg/l) were observed in 66.4% (*n*=506) and iron deficiency (ferritin < 35 μg/l) was observed in 19.8% (*n*=151) of participants, 2.1% (*n*=16) had ferritin < 12 μg/l.

**Figure 2:**
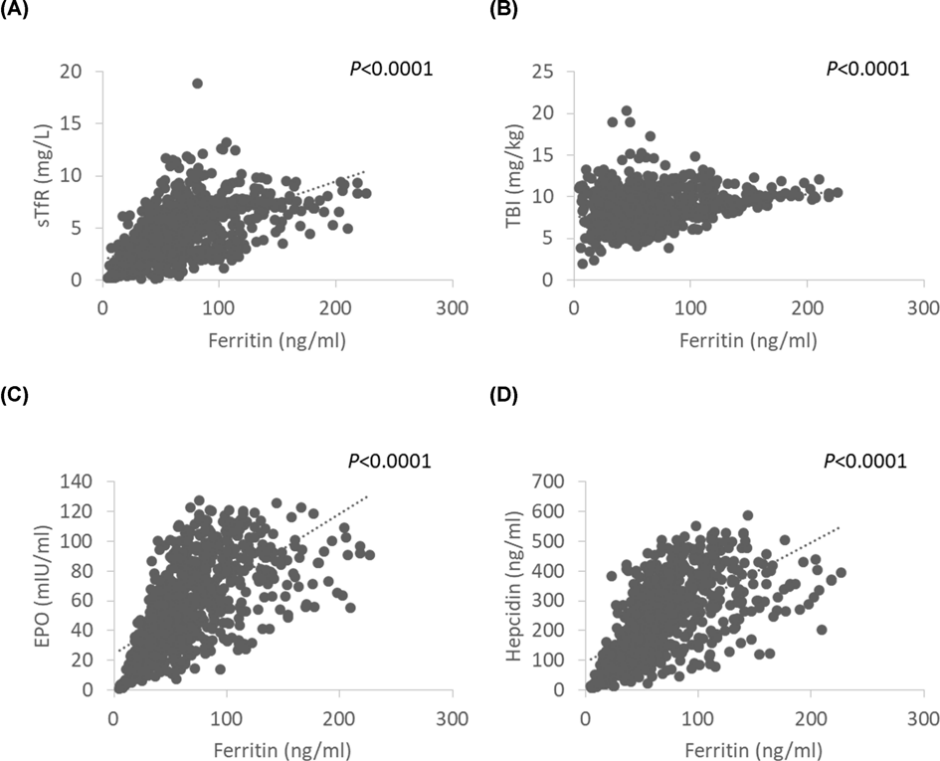
Association between different markers of iron stores in cord blood (**A**) Association between cord serum ferritin and cord serum sTfR (*n*=762). (**B**) Association between TBI estimates and cord serum ferritin (*n*=762). (**C**) Association between cord serum ferritin and cord serum EPO (*n*=762). (**D**) Association between cord serum ferritin and cord serum hepcidin level (*n*=762).

All subjects had estimated TBI values >0. In the present cohort, 7.5% (*n*=57) of subjects had sTfR levels >8.3 mg/l, the suggested threshold for iron-deficient erythropoiesis [17]. However, we found a positive correlation between serum ferritin and sTfR (r = 0.627, *P*<0.0001, Figure 2A). This suggests that the sTfR threshold level is not applicable to assess cord blood iron status. In addition, the calculated TBI values did not correlate well with cord serum ferritin (r = 0.262, *P*<0.0001) making TBI also questionable for evaluating in cord iron status (Figure 2B).

There was a strong positive correlation between cord ferritin levels and EPO (r = 0.696, *P*<0.0001, Figure 2C), as well as hepcidin (r = 0.686, *P*<0.001, Figure 2D).

## Discussion

The current study has investigated cord iron status using multiple approaches to estimate iron stores in normal birth weight, healthy, term infants at birth. We found a high prevalence of low iron stores in newborn cord serum samples, with 20% of newborns being iron deficient (cord serum ferritin levels <35 μg/l) and 67% having iron insufficiency (<76 μg/l). Two previous population-based studies performed in other parts of China reported a lower prevalence of iron insufficiency in cord compared with the findings of the present study. Shao et al. [7] performed a study of 3702 newborn infants in Hangzhou, reporting 9.5% of infants had ferritin levels <75 μg/l and only 1.4% had <35 μg/l. In Hebei province, Jones et al. [18] reported 26% of infants born to normal weight mothers (*n*=1084) had ferritin levels <75 μg/l, while 33% infants born to overweight mothers (*n*=233) had ferritin levels <75 μg/l. In other parts of the world, McCarthy et al. [8] reported 8% of a cohort of 413 Irish infants had low iron store (ferritin < 76 μg/l). In Norway, Hay et al. [19] reported 19% of 363 term infants had serum ferritin < 100 μg/l. In the U.S.A., in two high-risk cohorts (*n*=234, adolescents and multiple births) nearly 20% neonatal anemia was reported, but 40–50% of the mothers were also anemic [20,21].

This is the first study to report a positive association between cord serum ferritin and sTfR. Hay et al. [19] reported a negative association between ferritin and sTfR (r = −0.21, *P*<0.001) in 350 healthy term infants using an additive regression model. Sweet et al*.* [22] also reported a weak negative association between sTfR and ferritin (r = −0.23; *P*=0.007) in 144 cord blood samples. A negative association between sTfR and ferritin (r = −0.729; *P*<0.002) was also reported in a small Swedish cohort (*n*=15) of healthy term newborns [23]. Delaney et al. [9] also reported a negative association between sTfR and ferritin (r = −0.1318; *P*<0.05) in two high-risk U.S.A. cohorts. Other studies did not demonstrate an association between ferritin and sTfR concentrations in cord samples [24,25]. In adult populations, sTfR levels are negatively associated with serum ferritin concentrations, and elevated sTfR levels are an indicator of cellular iron insufficiency [11,12]. In the current study we report a positive association between ferritin and sTfR levels, it is not clear why these results differ from those previously reported but may reflect differences in gestational age range, ethnicity, analytical methodology, mode of delivery or length of time from birth to cord clamping. In the current cohort, we had a tightly controlled group of subjects, all were Han Chinese, in labor with vaginal delivery, without acute or chronic inflammation, and normal birthweight. While we only included term subjects (38+0 – 42 weeks gestational age) there was a positive association between gestational age and cord blood ferritin, sTfR, EPO, and hepcidin. In addition, while not found to be significantly associated with any of the studied markers of iron stores 6% of the subjects in the current study were clinically obese (BMI > 30) which may have an impact on the results [9,19,26]. Maternal stress during pregnancy may also impact neonatal iron stores [27]. Recent evidence also suggests that delaying cord clamping to allow continued flow of blood to the newborn is advantageous, particularly for preterm infants, but can also impact the measures of iron in the cord blood [28]. We therefore question the use of sTfR and subsequently TBI as indictors of iron status in young children, and particularly in neonates. Although TBI has been used and validated in adult populations as a useful indicator of body iron stores, it has not been used among infants and young children [10–12]. The current study has raised the need to further investigate the relationship between ferritin and sTfR among infants and young children, and more studies are required to evaluate the association between cord ferritin and sTfR before using TBI estimates to evaluate iron status in the newborns. Delaney et al*.* [9] have also questioned the application of current references ranges for use in neonates and how much they accurately reflect iron status.

In the current study, cord hepcidin correlated well with cord ferritin levels. When the body is iron replete, hepcidin concentrations are high, and iron supply to plasma is reduced; however, when iron demands are high, hepcidin concentrations are reduced, and more iron enters the circulation. Despite its crucial role in regulation of iron homeostasis, currently, little is known about fetal hepcidin and its regulatory function, and few normative hepcidin data are available. In the current study, although levels of inflammatory markers are low, we reported a much higher level of hepcidin than previously reported (49–110 ng/ml) [20,25,28], although there is currently no reference level/range available in newborn cord blood. In agreement with other studies we also reported cord hepcidin levels had a positive correlation with cord ferritin levels [28,29]. However, Kulik-Rechberger et al. [25] did not find any association between hepcidin and ferritin levels in cord blood and Rehu et al. [30] reported that cord serum hepcidin is independent of iron status in newborns. In addition, a study of monochorionic vs. di- or trichorionic pregnancies suggested that fetal hepcidin played a greater role in regulating fetal iron status than maternal hepcidin [21]. Understanding of hepcidin levels might provide insight into tissue demands for iron, however a lack of suggested threshold levels makes interpretation of hepcidin concentrations in terms of iron status difficult and of uncertain value. In addition, large variations in reported hepcidin levels also make it hard to interpret and its utility as an effective marker of iron status difficult to assess [20,21,25,28,30].

EPO is an endogenous human glycoprotein hormone whose main function is stimulating the production of hemoglobin. Little is known regarding EPO concentrations in newborns and its associations in response to insufficient iron status. This is the first study to evaluate the relationship between cord serum ferritin and EPO levels in a population-based study. In the present study, a positive association was found between EPO and ferritin. In addition, gestational age also positively predicted EPO levels. Lee et al. [20] also reported increased cord EPO levels with increasing gestational age, but they did not find an association between cord ferritin and EPO levels. However, Milman et al. [31] reported a strong negative association between EPO and ferritin in 90 term infants in Denmark (r = −0.54, *P*=0.0002). The levels of EPO measured in the present study are much higher compared with these other two studies.

One limitation of the study is that we did not also measure cord blood hemoglobin levels. In addition, we designed the study to investigate levels in apparently healthy, term, normal birth weight neonates from uncomplicated pregnancies. Investigation of other cohorts of newborns, from complicated or pre-term pregnancies, using the same methodology may be able to shed some light on the value of assessment of neonatal iron stores as well as potentially determining new reference ranges for newborns. Indeed, follow-up of the infants in the current study may also be useful to determine whether they stay iron insufficient or are able to rapidly increase their iron levels in the first few months of life. The high prevalence of insufficient iron stores identified here maybe a true reflection of the status of newborns in China, but may also reflect that reference ranges for different ethnicities need to be established. While we only included term subjects, there was still a positive association between markers of iron stores and gestational age, also suggesting that much tighter reference ranges, perhaps per week of delivery are required. While the current study may not add novel data to the literature, it does add further weight to the body of literature questioning the validity of current newborn reference ranges for assessment of iron stores, and their validity for assessing infant health.

In the current study we demonstrate a high prevalence of insufficient iron stores in a Chinese birth cohort. However, the value of cord sTfR and TBI in the assessment of iron status in the newborn is questionable, and new reference ranges may need to be established.

## Data Availability

All data are available from the corresponding author on request.

## Abbreviations

AGP: α1-acid glycoprotein
CRP: C-reactive protein
EPO: erythropoietin
GWCMC: Guangzhou Women and Children’s Medical Center
sTfR: soluble transferrin receptor
TBI: total body iron
